# *Rubus caesius* L. (European Dewberry) Extracts as a Novel Therapeutic Strategy Against MRSA Strains

**DOI:** 10.3390/ijms26146754

**Published:** 2025-07-14

**Authors:** Yahor Ivashchanka, Anna Hering, Alina Kastsevich, Justyna Stefanowicz-Hajduk, Rafał Hałasa

**Affiliations:** 1 Faculty of Pharmacy, Medical University of Gdansk, Al. Gen. J. Hallera 107, 80-416 Gdansk, Poland; egorivashka@gumed.edu.pl (Y.I.); kostevichalina@gumed.edu.pl (A.K.); 2 Department of Biology and Pharmaceutical Botany, Medical University of Gdansk, Al. Gen. J. Hallera 107, 80-416 Gdansk, Poland; anna.hering@gumed.edu.pl (A.H.); justyna.stefanowicz-hajduk@gumed.edu.pl (J.S.-H.); 3 Department of Pharmaceutical Microbiology, Medical University of Gdansk, Al. Gen. J. Hallera 107, 80-416 Gdansk, Poland

**Keywords:** antibiotics, DiSC_3_(5), leaf extract, NPN, stem extract, synergism

## Abstract

Increased bacterial resistance to current antibiotics leads to a depletion of therapeutic options in medicine. One of the problems of current therapy is methicillin-resistant Staphylococcus aureus (MRSA), which, in addition to resistance to β-lactam antibiotics, is multidrug-resistant. Some strains can also produce biofilms, a multicellular structure that is resistant or tolerant to various antibiotics. In hospitals worldwide, about 15% of invasive infections are caused by MRSA. Extracts of Rubus caesius (dewberry) contain high concentrations of compounds such as phenolic acids, flavonoids, tannins, and anthocyanins, which have potential antibacterial properties. This study is the first to demonstrate the activity of aqueous and ethanolic extracts of dewberry leaves (L_H2O_, L_EtOH_) and stems (S_H2O_, S_EtOH_) against S. aureus and Staphylococcus epidermidis. The most active extracts were L_EtOH_ (MIC 0.16 ± 0.40–1.56 ± 0.23 mg/mL) and L_H2O_ (MIC 0.16 ± 0.20–10 mg/mL). The study showed that L_EtOH_, S_EtOH_ and L_H2O_ extracts inhibited biofilm formation by clinical strains MRCN (methicillin-resistant coagulase-negative staphylococci) and MRSA (biofilm biomass reduction from 40 to 100%). Furthermore, 3,3′—dipropylthiacarbocyanine (DiSC_3_(5)) and N-phenyl-naphthylamine (NPN) were used to show that L_EtOH_ and S_EtOH_ caused the membrane depolarization of the strains studied. We also showed that the extracts acted synergistically and additively with cefoxitin and amikacin, reducing the MIC values of the antibiotics used by 8- to 16-fold and of the extracts tested by 4- to 8-fold. This study provides new data on potential antibacterial drugs of therapeutic importance.

## 1. Introduction

It has been over 90 years since the discovery of the first antibiotic, penicillin, by A. Fleming in 1928. Since then, many antibiotics have been developed. However, the frequent and unjustified use of antibiotics in healthcare and the food industry has led to rapidly spreading resistance among microorganisms. A group of important, antibiotic-resistant bacteria known as ESKAPE pathogens has been identified; Staphylococcus aureus is also included in this group [1,2].

*Staphylococcus* spp. is a Gram-positive bacteria that naturally inhabits the upper respiratory tract. More than 30% of the global healthy population is colonized by *Staphylococcus aureus* [3]. The bacteria mentioned can cause a range of infections, from folliculitis and abscesses to severe cases like sepsis. The pathogenicity of *S. aureus* strains depends on virulence factors present on the surface of bacterial cells, secreted enzymes, and toxins (e.g., hemolysins), staphylococcal enterotoxins (SEs), Panton–Valentine leukocidin (PVL), toxic shock syndrome toxin (TSST-1), and exfoliative toxins A and B (ETA and ETB) [3,4], which facilitate the spread of the bacteria in the human body [4]. In hospitals worldwide, approximately 15% of invasive infections are caused by methicillin-resistant *S. aureus* (MRSA) [3]. MRSA, in addition to being resistant to β-lactam antibiotics, are resistant to other groups of antibiotics, e.g., aminoglycosides and fluoroquinolones [5,6].

Moreover, some strains can produce a biofilm—a multicellular structure in which cells are embedded in a matrix. Biofilms are highly impermeable to chemicals; furthermore, they possess multiple mechanisms, making biofilm-associated infections particularly difficult to treat with conventional antibiotics [7].

In a biofilm state, MRSA is even more resistant than in a planktonic form, and it often causes infections associated with implants, catheters, and central lines. It is believed that around 80% of infections are related to biofilm-mediated infections caused by multi-resistant bacteria. Worldwide efforts are focused on finding highly effective and nontoxic alternatives with which to control MRSA biofilm infections [8]. One alternative approach to combating MRSA involves unconventional, safe-so-far, treatments derived from plant compounds [5,9,10]. Plants are an important source of antimicrobial compounds. Biologically active compounds present in plants include alkaloids, chalcones, tannins, flavonoids, and phenolic bioactive compounds [11].

Plants from the Rubus genus contain numerous biologically active compounds. To date, the influence of extracts from cloudberry fruit (*Rubus chamaemorus* L.) and raspberry (*Rubus idaeus* L.) on the development of biofilm and on the biofilm formed by MRSA strains has been studied [12]. Moreover, the effect of various extracts from the leaves of *R. fruticosus* L. on several bacteria, including *S. aureus* [10], and the inhibitory effect of *R. chingii* flower extract against multidrug-resistant bacterial strains, including S. aureus, was also demonstrated [13]. *S. aureus* was one of several bacteria tested for sensitivity to the essential oil from the leaves of *Rubus pungens* var. oldhamii [14], *Rubus idaeus* L. shoots [15], and the fruits from some cultivar varieties of *Rubus idaeus* and *Rubus occidentalis* [16].

Blackberries, especially *Rubus caesius* (dewberry), are among the most widely distributed blackberry species in Europe. The chemical composition of dewberry is well-documented, with high concentrations of compounds such as flavonoid, glycosides, aglycones, and anthocyanins, known for their antioxidant properties [17,18]. There are no reports on the antibacterial activity of dewberry leaves and stems.

In the presented study, our goal was to check whether extracts from *R. caesius* leaves and stems are active against bacteria of the *Staphylococcus* genus, including clinical MRSA strains and bacterial biofilm. The determination of the antibacterial mechanism of action of the extracts and the evaluation of its interactions with the antibiotics commonly prescribed to treat staph infections was also performed.

## 2. Results

### 2.1. Phytochemical Analysis of R. caesius Extracts

The phytochemical analysis of extracts *R. caesius* has been described by Hering et al. [17]. These include S_EtOH_—a stem ethanol extract; S_H2O_—a stem water extract; L_EtOH_—a leaf ethanol extract; and L_H2O_—a leaf water extract.

### 2.2. Determination of Minimum Inhibitory Concentrations (MIC) and Minimum Bactericidal Concentrations (MBC)

The antimicrobial potential of *R. caesius* was assessed against reference and clinical strains of bacteria. The extracts showed low inhibitory activity against MRCNS strains compared to gramicidin (300- to 3000-fold higher MIC values) but showed relatively high activity against MRSA strains (5- to 50-fold higher MIC values compared to gramicidin) (Table 1). Water extracts presented the lowest MIC against the test strains; MIC values were in the concentration range of 0.04 ± 0.15–>12.5 mg/mL. On the other hand, ethanol extracts showed lower MIC values compared to water extracts and were within the range of 0.16 ± 0.40–3.125 ± 0.45 mg/mL. In turn, leaf extracts showed similar MIC values against the tested strains, while the activity of stem extracts was varied. The bactericidal concentrations of the extracts were in the concentration range of 0.78 ± 0.41–>12.5 mg/mL. For most of the tested strains, the least active turned out to be S_H2O_ extracts (MBC values were >12.5 mg/mL). The remaining extracts showed a similar level of activity against bacteria (MBC 0.78 ± 0.41–>10 mg/mL). It should be noted that clinical strains showed higher sensitivity (lower MIC and MBC values) to the extracts (S_EtOH_, L_EtOH_, L_H2O_) than the reference strains.

The antibacterial activity of the extracts was determined by the microbroth dilution method. The results show (Table 1) that L_EtOH_ —leaf ethanol extract—and L_H2O_—leaf water extract—are characterized by a similar level of bacteriostatic (MIC 0.16 ± 0.20–10 ± 0.54 mg/mL) and bactericidal activity (MBC 0.78 ± 0.48–12.5 ± 0.54 mg/mL) against the reference and clinical strains of *Staphylococcus* spp. The tested extract S_EtOH_ —stem ethanol extract (MIC 0.16 ± 0.40–3.125 ± 0.45 mg/mL; MBC 0.78 ± 0.41–12.5 ± 0.65 mg/mL)—was weaker in terms of activity, and the least active turned out to be the extract S_H2O_—stem water extract (MIC 0.04 ± 0.15– > 12.5 mg/mL; MBC > 12.5 mg/mL). Notably, clinical strains (MRSA; MRCNS) showed higher sensitivity in terms of the extracts (S_EtOH_, L_EtOH_, L_H2O_) than the reference strains.

### 2.3. Inhibition of Biofilm Formation

**Figure 1:**
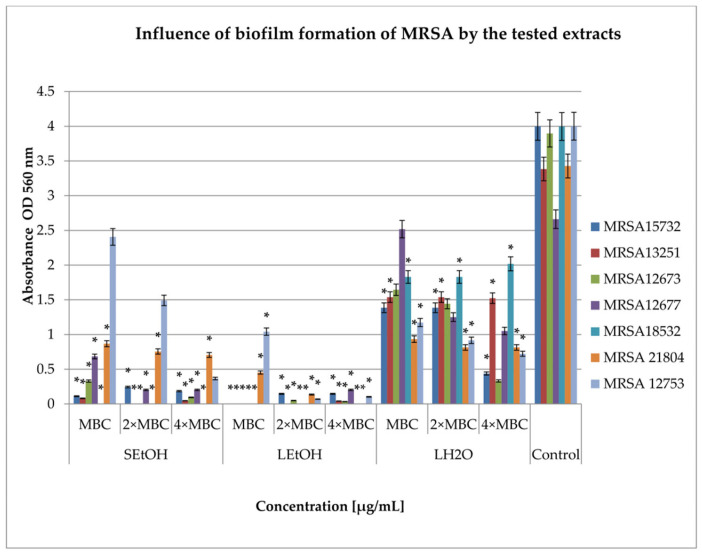
Influence of *R. caesius* extracts on biofilm formation of MRSA strains. Control group was bacterial sample without compounds. Minimum bactericidal concentration—MBC; double minimum bactericidal concentration—2 × MBC; quadruple minimum bactericidal concentrations—4 × MBC. Results are presented as mean values ± standard deviation (±SD) from three independent experiments. Error bars represent standard deviation. (*) *p* < 0.05 was considered statistically significant. Control consisted of strains grown without extracts.

**Figure 2:**
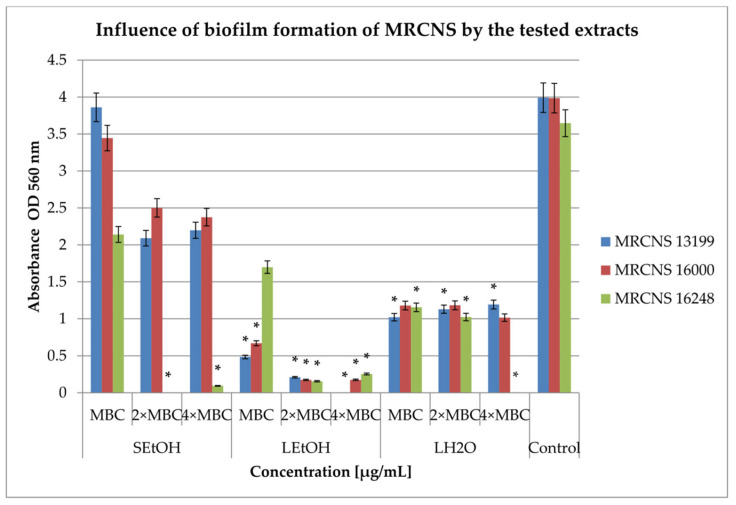
Influence of *R. caesius* extracts on biofilm formation of MRCNS strains. Control group was bacterial sample without compounds. Minimum bactericidal concentration—MBC; double minimum bactericidal concentrations—2 × MBC; quadruple minimum bactericidal concentrations—4 × MBC. Results are presented as mean values ± standard deviation (±SD) from three independent experiments. Error bars represent standard deviation. (*) *p* < 0.05 was considered statistically significant. Control consisted of strains grown without extracts.

In this study, two key mechanisms were analyzed: the inhibition and eradication of bacterial biofilm by the extracts. The S_H2O_ extract did not show bactericidal properties in the range of concentration. As such, for the remaining studies, we used only three extracts, S_EtOH_, L_EtOH_, and L_H2O_. The extracts were tested at three concentrations: MBC, 2 × MBC, and 4 × MBC. The results showing the effect of the tested extracts on biofilm formation by clinical strains are presented in Figure 1 and Figure 2.

The study results (Figure 1) show that the highest degree of inhibition of biofilm formation was observed in the case of L_EtOH_, ranging from 74% for the MRSA12753 strain (MBC concentration; *p* < 0.05) to 100% for the MRSA18532 strain (all tested concentrations; *p* < 0.05). The S_EtOH_ extract caused a reduction in biofilm formation from 40% against the MRSA12753 strain (MBC concentration) to 100% against the MRSA18532 strain (all tested concentrations; *p* < 0.05). The L_H2O_ extract showed a varying degree of reduction, ranging from 5% against the MRSA12677 strain (MBC concentration) to 91% against the MRSA12677 strain (4 × MBC concentration; *p* < 0.05).

The effects of the extracts on the biofilm formation of MRCNS (Figure 2) revealed that leaf extracts were more active than stem extracts. Moreover, the highest biofilm inhibition concentrations were achieved for L_EtOH_ extract—from 53% against MRCNS16248 (MBC concentration) to 100% against MRCNS 13199 (4 × MBC concentration; *p* < 0.05). In turn, L_H2O_ extract showed a degree of reduction from 68% against MRCNS 16248 (MBC; *p* < 0.05) to 100% against MRCNS 16248 (4 × MBC; *p* < 0.05). The least active, S_EtOH_ extract, inhibited biofilm formation from 3% on MRCNS13199 (MBC concentration) to 100% on MRCNS16248 (2 × MBC concentration; *p* < 0.05).

### 2.4. Influence on the Biofilm Formed

**Figure 3:**
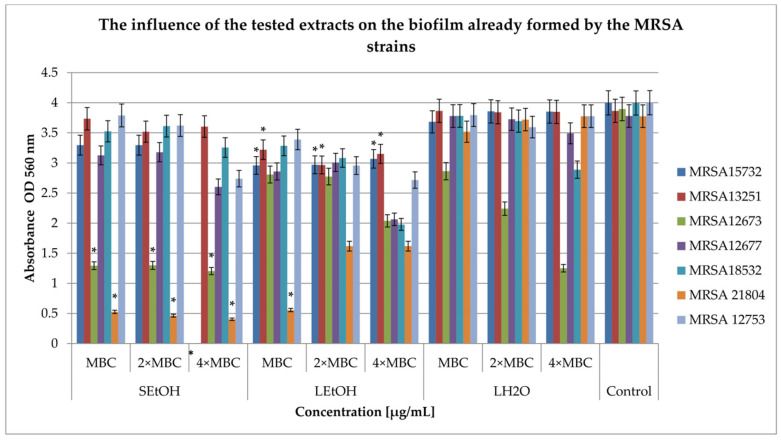
Influence of *R. caesius* extracts on MRSA pre-formed biofilm. Control group was bacterial sample without compounds. Minimum bactericidal concentrations—MBC; double minimum bactericidal concentrations—2 × MBC; quadruple minimum bactericidal concentrations—4 × MBC. Results are presented as mean values ± standard deviation (±SD) from three independent experiments. Error bars represent standard deviation. (*) *p* < 0.05 was considered statistically significant. Control consisted of strains grown without extracts.

**Figure 4:**
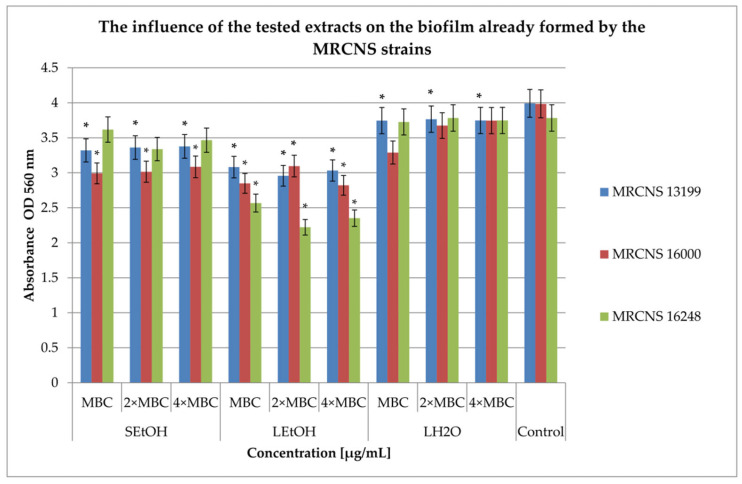
Influence of *R. caesius* extracts on MRCNS pre-formed biofilm. Control group was bacterial sample without compounds. Minimum bactericidal concentrations—MBC; double minimum bactericidal concentrations—2 × MBC; quadruple minimum bactericidal concentrations—4 × MBC. Results are presented as mean values ± standard deviation (±SD) from three independent experiments. Error bars represent standard deviation. (*) *p* < 0.05 was considered statistically significant. Control consisted of strains grown without extracts.

Studies on the effect of three extracts on the formed biofilm were carried out at three concentrations: MBC, 2 × MBC, and 4 × MBC. The results for MRSA and CNS strains are presented in Figure 3 and Figure 4, respectively.

Figure 3 shows the effect of extracts on MRSA strains in the formed biofilm. The impacts of various impacts on the development of biofilm of MRSA strains were observed. The S_EtOH_ extract inhibited further biofilm growth in the range from 3% (MBC concentration) against the MRSA13251 strain to 100% (4 × MBC; *p* < 0.05) against the MRSA15732 strain. The L_EtOH_ extract inhibited biofilm development from 15% against the MRSA12753 strain (MBC concentration) to 57% against MRSA 21804 (concentration 2 × MBC and 4 × MBC; *p* < 0.05). In turn, the L_H20_ extract was not active against the MRSA 12677 strain in the MBC concentration, and against the MRSA 13251 strain in all tested concentrations. In addition, its activity was demonstrated in inhibiting the development of biofilm from 1% against the MRSA12677 and 21804 strains in a concentration of 2 × MBC to 67% against MRSA 12673 in a concentration of 4 × MBC.

In the case of the biofilm formed by the MRCNS strains (Figure 4), the antibacterial effect of the extracts was weaker compared to the studies conducted on MRSA strains. The L_EtOH_ extract inhibited the development of biofilm, with inhibition ranging from 22% in the MBC concentration against MRCNS1600 and 13199 (*p* < 0.05) to 41% against MRCNS16248 in a concentration of 4 × MBC (*p* < 0.05). The tested concentrations of the S_EtOH_ extract inhibited further biofilm development, from 4% against the MRCNS 16248 strain (at MBC concentrations) to 24% against the MRCNS16000 strain (4 × MBC; *p* < 0.05). The L_H2O_ extract was the least active and did not show any inhibitory effect against the MRCNS16248 strain (at concentrations of 2 × MBC and 4 × MBC). Against the remaining strains, it inhibited biofilm formation in the range of 1% for the MRCNS16248 strain (at MBC concentrations) to 17% for the MRCNS1600 strain. The extract for the MRCNS13199 strain resulted in statistically significant changes in the amount of biofilm (*p* < 0.05).

### 2.5. Membrane Depolarization Assay

To assess the mechanism of action of blackberry extracts and to determine whether the mechanism is related to the depolarization of bacterial membranes, we performed a study of the effects on membrane potential. The cytoplasmic membrane depolarization ability of the extracts was analyzed using a sensitive dye, 3,3′-dipropylthiacarbocyanine (DiSC_3_(5)). Gramicidin at a concentration of 125 µg/mL was used as a positive control. The results are summarized in Table 2.

Of all the extracts tested, the depolarization effect was demonstrated by ethanol extracts from stems (from about 62% against MRSA15732 to 298% against *S. aureus* ATCC6538) and leaves (from 73% against MRSA15732 to 165% against *S. aureus* ATCC6538). However, the L_EtOH_ extract did not demonstrate such a mechanism against MRCNS13199. L_H2O_ and S_H2O_ extracts did not provide the expected mechanism of action. Based on the results obtained, it can be concluded that ethanol extracts from blackberry stems and leaves disturbed the membrane potential of bacterial cells. Water extracts do not demonstrate this effect.

### 2.6. Membrane Integrity Assay

**Figure 5:**
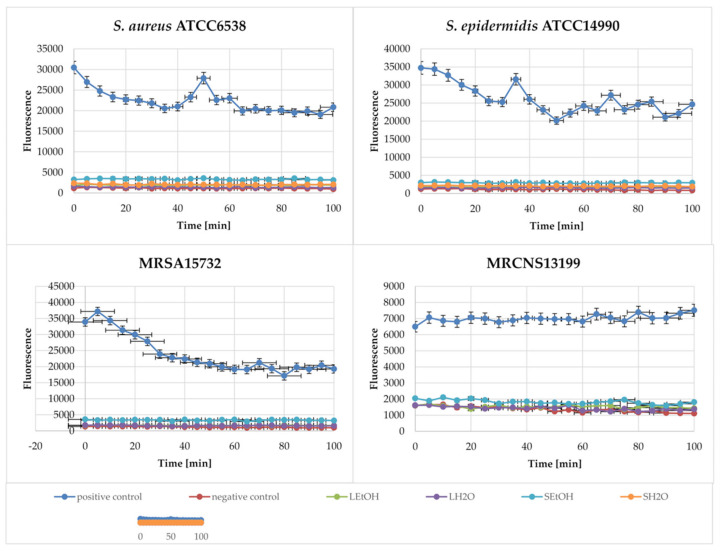
NPN membrane integrity assay for selected bacterial strains and *R. caesius* extracts. Positive control: gramicidin at a concentration of 125 µg/mL. Negative control: bacterial strain. Results are presented as mean values ± standard deviation (±SD) from three independent experiments. Error bars represent standard deviation.

A study using N-phenyl-naphthylamine (NPN), which does not show fluorescence in solution but causes it after entering the hydrophobic environment (the ruptured bacterial cell membrane), was conducted [19]. Gramicidin at a concentration of 125 µg/mL was used as a positive control. The results of the tests are presented in Figure 5.

The presented results (Figure 5) show a high level of fluorescence in the case of the positive control, gramicidin. However, the extracts did not cause the rupture of the cell membrane in the cells of the bacterial strains, which is visible from the close-to-zero level of fluorescence of the marker present in the samples. A slightly higher level of fluorescence was noted in the samples where the MRCNS13199 strain was used.

### 2.7. Extract–Antibiotic Synergy Against MRSA

The synergistic action of antibiotics and extracts is calculated based on determination of the MIC value for the extract, antibiotic, and the combination of both components. Based on the MIC values, it is possible to determine the FICI (Fractional Inhibitor Concentration Index) and demonstrate the interactions between the tested extracts and antibiotics.

To study the interactions between extracts and antibiotics, amikacin and cefoxitin were selected. The studies were carried out using strains MRSA12673 and MRSA15732, which are characterized by a high level of biofilm production and sensitivity to extracts. The results of the studies are included in Table 3.

Then, FICI was interpreted as follows: synergism (FICI < 0.5), semi-synergism (0.5 < FICI < 1.0), additive effect (1 < FICI < 4), and antagonism (FICI > 4)

The extracts showed various interactions with the antibiotics used for the tested strains. L_EtOH_ extracts with amikacin showed synergism for the MRSA12673 strain and partial synergism for the MRSA15732 strain. A synergistic effect was also observed in the case of the combination of L_EtOH_ and S_EtOH_ extracts and cefoxitin for the MRSA12673 strain. Similarly, L_EtOH_ and L_H2O_ extracts in combination with cefoxitin acted synergistically for the MRSA15732 strain. L_H2O_ and S_EtOH_ extracts showed partial synergism with cefoxitin against MRSA12673 and MRSA15732, respectively. Additivity was demonstrated by the combination of amikacin with S_EtOH_ and cefoxitin with S_H2O_ for both tested strains. Amikacin combinations with S_H2O_ and L_H2O_ showed antagonistic activity against both strains.

## 3. Discussion

The decreasing number of active antibiotics in the fight against human infections caused by multidrug-resistant bacteria forces us to search for new biologically active compounds. Secondary metabolites of plants are a good source of antibacterial compounds. Plants of the *Rubus* genus are particularly interesting because they are a promising source of biologically active compounds. The study of the antibacterial activity of *Rubus caesius* extracts shows that leaf extracts (ethanolic and aqueous) are active in demonstrated concentrations from 0.16 ± 0.20–10 ± 0.54 mg/mL (MIC), while stem extracts (ethanolic and aqueous) are active in concentrations from 0.04 ± 0.15 to >12.5 mg/mL (MIC). Interestingly, resistant staphylococci strains turned out to be more sensitive to the extracts than the antibiotic-sensitive reference strains.

Previous studies [17] focused on other bacterial species and showed the high activity of the tested *Rubus caesius* extracts against *Clostridium sporogenes* ATCC19404 and *Clostridium bifermentans* ATCC638 (MIC 0.03125–0.5 mg/mL), while the activity was much weaker against Gram-negative bacteria. Grabek-Lejko et al. [20], using a well diffusion method, studied the effect of crude extracts from fruits and leaves of blackberries (*Rubus fruticosus*) and raspberries (*Rubus idaeus*). The authors showed that fruit extracts were more active than leaf extracts at a concentration of 10 mg/mL against the tested bacterial strains. Simultaneously, they showed that the extracts were more active against *S. aureus* ATCC25923 than against *E. coli* PCM2561 [21]. In turn, Welia et al. [11], using the disc diffusion method, showed that only the hydro alcoholic extract from *Rubus fruticosus* leaves inhibited the growth of *S. aureus* (250–2000 µg/mL). Using the same method for the evaluation of antibacterial activity, Krzepiło et al. [22] studied the effect of extracts of leaf buds of several varieties of raspberries *Rubus idaeus* L., thornless blackberry *Rubus fruticosus* L., blackberry *Rubus fruticosus* L, and a mixture of *Rubus idaeus* L. and *Rubus fruticosus* L. They showed that all extracts were effective against *S. aureus*. Other studies [Grabek-Lejko [23] presented that methanol–water (50:50, *v/v*) extracts from blackberry (*R. plicatus*) and raspberry (*R. idaeus*) fruit and leaves showed a similar level of activity against *S. aureus* ATCC25923 (MSSA), *S. aureus* ATCC43300 (MRSA), and two clinical strains.

Studies of ethanol extracts from different parts of *Rubus ulmifolius* presented that the extract from roots and woody stems showed antibacterial activity against *S. aureus* ATCC33593 (MIC_50_—512 µg/mL), while extracts from leaves, stems, and flowers showed no activity in the tested concentration range. Studies conducted using the extract from *Rubus ulmifolius* Schott. roots (fraction 220D-F2) against 15 clinical strains of *S. aureus* showed that MIC_90_ values ranged from 530 to 1040 µg/mL, and MBC_90_ ranged from 530 to 2040 µg/mL. The analysis of fraction 220D-F2 showed that it mainly contained ellagic acids (EA) and ellagic acid derivatives (spogenin-related compounds; EADs) [24,25]. In general, it can be said that extracts from various blackberry species are characterized by high activity against various strains of S. aureus, including MRSA, which was also demonstrated in the presented studies.

The differences in activity presented by individual authors are related to extraction procedures, the type of solvent used, the geographical origin of plants, and the time of harvesting of the plant parts [23].

Various studies have shown that the activity of herbal extracts may be related to the presence of biologically active ingredients. Using LC-ESI-MS/MS with MRM analysis, flavonoids were isolated and determined [17], and it should be noted that there were visible differences between the quantitative composition of individual compounds in water and ethanol extracts. Water extracts (S_H2O_, L_H2O_) were characterized by significantly lower amounts of flavonoids than ethanol extracts (S_EtO_, L_EtO_). Moreover, the ethanol extracts contained significant amounts of hyperoside (5–10-fold more than the water extracts), tiliroside (L_EtOH_ contained 4-fold more than S_EtOH_), isoquercetin, rutin, quercetin, and isokaempferide, whereas, in the S_EtOH_ extracts catechins were found (3.5-fold more than L_EtOH_). The L_H2O_ activity may be related to biologically active compounds that have not yet been identified. The presence of catechins is often associated with antimicrobial activity and their mechanism is related to interactions with the cell membrane [26,27].

Sanver et al. [28] showed that rutin (quercetin-3-O-rhamnoglucoside), quercetin, and tiliroside affect the structure of the cytoplasmic membrane. It is also known that, among others, quercetin inhibits 3-hydroxyacyl-ACP dehydrase from *Helicobacter pylori* [29], an enzyme involved in bacterial type II fatty acid synthase (FAS-II). Moreover, quercetin can affect enzymes involved in the biosynthesis of the peptidoglycan of the bacterial cell wall [30] and binds to the B subunit of DNA gyrase from *E. coli*, *Mycobacterium smegmatis*, and *M. tuberculosis* [27,31]. It is also known that quercetin, quercetin-3-glucoside (isoquercetin), and quercetin-3-O-rhamnoside (quercitrin) prevent ATP hydrolysis in bacterial cells but do not affect ATP synthesis [32]. Perhaps hyperoside (quercetin 3-D-galactoside), as a derivative of quercetin (present in significant amounts in S_EtOH_ and L_EtOH_), has a similar effect. Furthermore, the hydrophilic nature of quercetin limits its penetration into the bacterial cell [27]. Therefore, it was necessary to check the mechanism of action of whole extracts on bacterial cells.

The assessment of the effect of extracts on biofilm-forming bacteria constituted the next stage of the work. Biofilm is a multicellular structure surrounded by a polymeric extracellular matrix that protects bacteria from chemicals and other stressors, making it difficult to treat infections in which biofilm may be present. This highlights the urgent need to develop effective treatments for such infections. The inhibition of biofilm formation is crucial due to the ability of bacteria to form biofilm layers on medical devices such as endotracheal tubes and catheters, leading to severe and chronic infections in the respiratory, urinary, and circulatory systems [33].

The activity of extracts against MRSA and MRCNS biofilms was studied in two groups. The first group included studies on the effect of extracts on biofilm formation by the tested strains, while the second one focused on the assessment of the growth inhibition of already formed biofilm. The L_EtOH_ extract turned out to be a highly effective inhibitor of biofilm formation, active against MRSA and MRCNS strains.

The S_EtOH_ extract had a very strong effect on MRSA strains, while MRCNS strains were less sensitive. The opposite effect was observed for the activity of the L_H20_ extract, which was active against MRCNS strains but was less active against MRSA strains.

In turn, a complete lack of activity was demonstrated against the tested strains by the S_H2O_ extract. The extracts we tested were characterized by significantly weaker activity against staphylococcal strains in an already formed biofilm. It should be added here that the presented studies were conducted using crystal violet, which shows the overall level of biofilm biomass under the influence of compounds, but does not track the effect of the tested extracts on the bacterial cells themselves present in the biofilm. It can be hypothesized that the large effect of the extracts we tested on the stage of biofilm formation may be related to the inhibition of communication between bacteria via the quorum sensing system. Quorum sensing, a bacterial communication system reported in various bacterial strains that regulates biofilm formation, is mediated by autoinducers [34]. Citrus flavonoids, including apigenin, kaempferol, quercetin, and naringenin, present in the tested ethanol extracts (S_EtOH_ and L_EtOH_) were found to be effective antagonists of quorum sensing signaling [34]. Quercetin was found to inhibit N-acyl-homoserinolactone (AHL)-dependent alginate and QS production. Furthermore, quercetin increases the expression of genes for several siderophores that capture Fe^3+^, which is required for Pseudomonas aeruginosa biofilm formation [35,36].

Another group of researchers [24] conducted studies on the extract of Rubus ulmifolius Schott. roots and isolated fraction 220D-F2. This fraction was very active in preventing biofilm formation against 15 genotypically diverse clinical isolates of *S. aureus* (MBIC_90_ 50–200 µg/mL) [24]. In the studied fraction, the authors identified two new compounds: EA rhamnoside, and EA xyloside. The EA xyloside inhibited S. aureus growth and biofilm formation (MIC_50_ (32 µg/L) more than it did MBIC_50_ (64 µg/mL)). Only EA rhamnoside was able to inhibit biofilm formation by about 90% [37]. The authors suggest that sugars are a determinant of the biological activity of the extracts studied, but this effect is very diverse. In the case of the results presented in the manuscript, it can be concluded that quercetin and its derivatives can affect the biofilm produced by MRSA strains.

Information on the effect of herbal extracts on bacterial cells, affecting the integrity of the cytoplasmic membrane, is available in most publications examining the antimicrobial activity of plant extracts [18]. The presented studies focused on understanding the above-mentioned mechanism, including the study of membrane depolarization (DiSC_3_(5)) and the study of membrane integrity (NPN). High depolarization activity was demonstrated by the S_EtOH_ extract, which may have resulted from the simultaneous presence of catechins, rutin, and hyperoside (quercetin 3-D-galactoside). The effect of the tested extract was higher for the MRCNS strain than for the MRSA strain. On the other hand, the L_EtOH_ extract exerted a membrane depolarization effect, most likely related to the presence of hyperoside, rutin, tiliroside, and catechin [17]. Unfortunately, this activity of the L_EtOH_ extract was weaker, perhaps due to the three-fold lower content of catechins and the smaller amount of rutin. The extract was effective against the MRSA strain, but it did not work against the MRCNS strain. In turn, the tested aqueous extracts did not cause membrane depolarization, which was probably caused by the lack of catechins and small amounts of rutin, hyperoside, tiliroside, and quercetin in their composition. However, we did not find that the extracts we tested showed a bactericidal effect through the integration/disruption of cytoplasmic membranes, which was attributed to the designated extract components.

One group of researchers showed that the mechanism of action of catechins is based on interaction with the bacterial cell membrane. This mechanism is visible in the presented studies. In the other literature sources, it can be found that the mechanism of action of catechins involves the disruption of the cell membrane by binding to the lipid bilayer and via the inactivation or inhibition of the synthesis of intracellular and extracellular enzymes [26]. Such a mechanism is not visible in the results of the studies presented in the manuscript. Cushnie et al. [38] examined the activity of catechins and found that the disruption of the cell membrane causes potassium leakage in the *Staphylococcus* MRSA strain, which is the first indicator of membrane damage in microorganisms (Lambert). In turn, Sanver et al. [28] showed that quercetin, rutin (quercetin-3-O-rhamnoglucoside), and tiliroside reduced the thickness of the cytoplasmic membrane bilayer. Moreover, it was also shown that rutin disrupted the structure of the lipid layer of the bacterial cytoplasmic membrane.

The studies presented in the manuscript focused on the therapeutic use of the extracts obtained in the fight against microorganisms causing infections in humans. High activity against *S. aureus* ATCC6538 and *S. epidermidis* ATCC14400 prompted us to investigate resistant strains, including the study of extract–antibiotic interactions in order to obtain better therapeutic effects. Attempts to demonstrate interactions between extracts and antibiotics focused on amikacin (aminoglycosides) and cefoxitin (beta-lactams). The results obtained showed that the L_EtOH_ extract exerted a synergistic effect with amikacin and cefoxitin. The same effect was observed for S_EtOH_ extract and cefoxitin, but S_EtOH_ acted additively with amikacin. In turn, water extracts presented antagonistic effects with amikacin and synergistic (L_H2O_) or additive (S_H2O_) with cefoxitin. Other researchers [10] studied the interactions of blueberry and blackberry pomace extracts (BPEs) with methicillin against MRSA and showed a reduction in the MIC for methicillin from >512 µg/mL to 4 µg/mL in combination with different concentrations of BPE.

The agar diffusion method was used to screen the interactions of extracts from *R. idaeus* “Willamette” shoots (rich in ellagitannins, free and conjugated ellagic acids, epicatechin, and quercetin 3-O-glucuronide) and extracts from *R. idaeus* and *R. occidentalis fruits* (composed of flavonoids, ellagitannins, phenolic acids, and anthocyanins) with selected antibiotics against *S. aureus* ATCC6538 and *S. epidermidis* ATCC14990 [39]. The extracts showed synergism with penicillin and oxacillin (beta-lactam antibiotic) against *S. aureus* and antagonism with antibiotics from the aminoglycoside group. In the case of *S. epidermidis*, most of the interactions between the antibiotics used and the extracts were found to be antagonistic. The results presented in the manuscript are consistent with the results presented in the literature.

The presence of quercetin and its derivatives in the extracts tested may cause a synergistic effect with cefoxitin. Similar interactions have been detected with other beta-lactam antibiotics (ampicillin, cephradine, ceftriaxone, imipenem, or methicillin) [40]. Quercetin and amoxicillin showed synergistic effect against MDR *S. epidermidis* by reducing the content of fatty acids and increasing the permeability of the cytoplasmic membrane, the inhibition of peptidoglycan synthesis, and the activation of β-lactamase [41,42]. In turn, epicatechin presented synergistic activity against clinical strains of MRSA with the following beta-lactam antibiotics: ampicillin, ampicillin/sulbactam, cefepime, cefazolin, and imipenem/cilastatin [42,43]. It has been shown that (-)-epigallocatechin gallate acted against clinical and reference strains of MRSA and MSSA, affecting virulence factors; impacted the cell wall; and showed synergy with penicillin, oxacillin, imipenem, ampicillin/sulbactam, banipenem, meropenem, oxytetracycline, and tetracycline [27,44,45,46,47,48,49]. In the case of synergism with beta-lactams, apigenin and quercetin may demonstrate another mechanism action—the inhibition of D-alanine-D-alanine ligase, an enzyme responsible for the synthesis of the peptidoglycan precursor of the bacterial cell wall [30]. The mechanism of synergy of the extracts studied in the manuscript with cefoxitin may be a combination of ligase inhibition by apigenin and quercetin and the simultaneous inhibition of BPBs (penicillin-binding proteins) activity by cefoxitin.

In turn, it has been shown that isoquercetin exhibited antagonistic effects with aminoglycoside antibiotics, e.g., neomycin, kanamycin, gentamycin, and amikacin, against *E. coli* strains. On the other hand, it was noted that isoquercetin and quercetin did not affect the activity of aminoglycosides against multi-resistant *S. aureus* strains [18,40,50]. Quercetin (and its derivatives) and epicatechin [40] have been shown to inhibit F1F0-type ATP synthase, a key enzyme in bacterial cellular energy metabolism [40]. This enzyme uses a proton gradient and the associated membrane potential to synthesize ATP. It can also hydrolyze ATP, creating a proton gradient, which can be used to transport aminoglycosides into the bacterial cell. Disturbances in ATP synthase activity may cause antagonistic interactions of the extracts tested with amikacin. In the presented studies, antagonism with amikacin was demonstrated using aqueous extracts and it was very difficult to determine which of the known components caused such an effect of interactions.

The action mode of the extracts on the bacterial cell is complex, and the lack of full data on the composition of the extracts makes it difficult to identify the components responsible for the visible effects. Therefore, to precisely explain the mechanisms of action, it is necessary to know the composition of the extracts well. However, based on the composition known so far and on the results obtained, it is possible to conclude that the extracts, especially L_EtOH_ and S_EtOH_, can give the expected therapeutic effect (i) independently, as compounds disrupt communication between microbial cells (quorum sensing)—which is a very important element of the bacterial biofilm formation process on artificial materials, e.g., central punctures, but also on surgical wounds—and (ii) in combination with antibiotics, acting synergistically and reducing the effective dose of the drug that can be used. This, in turn, will reduce possible side effects of the drug and the cost of treatment for the patient. However, further analyses of the composition of the extracts should be carried out. Next, it is important to determine the effect of synergy on biofilm, demonstrate the effect on cells in biofilm, and evaluate the rate of resistance to extracts and its combinations with antibiotics development.

## 4. Materials and Methods

### 4.1. Materials

#### 4.1.1. Extract Preparation

Preparation methods and phytochemical analysis of *R. caesius* extracts have been described previously by Hering et al. [17].

#### 4.1.2. Bacteria

*Staphylococcus aureus* ATCC6538, *Staphylococcus epidermidis* ATCC14990, and methicillin-resistant clinical strains (*Staphylococcus aureus* MRSA18532, MRSA13251, MRSA12673, MRSA12677, MRSA15732, MRSA21804, MRSA12753, methicillin-resistant clinical strain coagulase-negative *Staphylococcus* sp. MRCNS16000, MRCNS16248, MRSE13199) were sourced from the collection of the Department of Pharmaceutical Microbiology, Medical University of Gdańsk.

#### 4.1.3. Materials and Growing Conditions

Gramicidin is amixture of gramicidins A, B, C, and D (Sigma-Aldrich, Merck Life Science Sp. z o.o., Darmstadt, Germany), cefoxitin sodium salt (Pol-Aura, Zawroty, Poland), amikacin disulfate salt (Pol-Aura, Zawroty, Poland), N-phenyl-1-naphthylamine (NPN) (Termo Fisher Scientific, Waltham, MA, USA), and 3,3-dipropylthiacarbocyanine (DiSC_3_(5)) (Termo Fisher Scientific, Waltham, MA, USA).

Mueller–Hinton broth (MH, Becton Dickinson, Franklin Lakes, NJ, USA) was used for MIC and FICI determination. Tryptic soy broth (TSB, Becton Dickinson Franklin Lakes, NJ, USA) medium supplemented with 2% glucose was applied in order to culture the biofilm bacteria. Bacteria were cultured in an aerobic atmosphere at 37 °C for 24 h. MH agar plates were used to determine bacterial viability.

### 4.2. Determination of Minimum Inhibitory Concentrations and Minimum Bactericidal Concentrations

Minimum inhibitory concentrations (MICs) were determined by the microdilution method as described by Krazue-Baranowska et al. [16]. Initially, test samples were diluted in sterile water (50 mg/mL). Next, Mueller–Hinton broth was added to each well, and then test samples were added to the first wells to reach a final volume of 100 µL. Samples were diluted according to a geometric concentration progression from 12.5 mg/mL in the first well to 0.0061 mg/mL. An overnight bacterial culture in Mueller–Hinton broth was suspended in phosphate-buffered saline (PBS) to reach a final concentration of 0.5 × 10^6^ CFU/mL. Plates were incubated at 37 °C for 24 h. The MIC was defined as the lowest concentration at which no visible growth was observed. Assays were performed in triplicate.

To determine the MBC, the contents of each well were plated on MH agar. The plates were incubated at 37 °C for 24 h, and the MBC values were determined. The MBC was defined as the lowest concentration at which no bacterial growth was observed. Assays were performed in triplicate.

### 4.3. Influence of Extracts on Biofilm Formation and on Pre-Formed Biofilm

The previously described methodology [50] was used with some modifications. The MIC concentrations of extracts were replaced by MBC concentrations. Measurements were performed using a microplate reader (Infinite^®^ 200 PRO, Tecan, Männedorf, Switzerland).

### 4.4. 3,3-dipropylthiacarbocyanine (DiSC_3_(5) Assays

The ability of *R. caesius* extracts to depolarize the cytoplasmic membrane of *Staphylococcus* spp. was determined using the membrane potential-sensitive dye DiSC3(5) [51,52,53]. The dye itself does not exhibit fluorescence in solution. However, it easily penetrates cells, where it remains until the membrane potential is disrupted. Once the potential is disturbed, the dye passes into the solution and causes measurable fluorescence [19]. Initially, bacterial suspensions were prepared as follows: mid-logarithmic-phase bacterial cells were collected by centrifugation in 5 mL Eppendorf tubes (4200 rpm for 15 min.), washed twice with PBS buffer, and diluted to approximately 1.5 × 10^8^ CFU/mL in the same buffer. The cell suspension was initially incubated with 200 µM KCl at room temperature for 30 min, then with 0.7328 µM DiSC_3_(5) for an additional 30 min in the dark. After incubation, 1 × MBC concentrations of the extracts, positive controls (125 µg/mL of gramicidin S; MBC), and blank controls (PBS instead of extracts) were transferred into 96-well plates. Then, the bacterial suspension was added to the respective wells. The fluorescence intensity was monitored in real time using a microplate reader (Infinite^®^ 200 PRO, Tecan, Männedorf, Switzerland), with the filter set to an excitation wavelength of 622 nm and an emission wavelength of 670 nm. Membrane depolarization assays were performed in triplicate (n = 3) and the membrane potential-dissipating activity of the extracts was calculated as follows [4]:

% membrane depolarization = 100 × [(F_p_ − F_0_)/(F_g_ − F_0_)],

where F_0_ denotes fluorescence of the blank, F_p_ denotes the fluorescence signal 30 min after addition of extracts, and F_g_ denotes the fluorescence signal after addition of gramicidin S.

### 4.5. N-phenyl-1-naphthylamine (NPN) Assays

Outer membrane permeabilization was studied using an N-phenyl-1-naphthylamine, as described previously [19,54], with some modifications. Initially, bacterial suspensions were prepared as follows: mid-logarithmic-phase bacterial cells were collected by centrifugation in 5 mL Eppendorf tubes (4200 rpm for 15 min), washed twice with PBS, and diluted to approximately 1.5 × 10^8^ CFU/mL in PBS. The NPN was added to the suspension in a concentration of 10 µM. Then, a 96-well plate was prepared by adding 1 MBC concentrations of the extracts, positive controls (125 µg/mL of gramicidin S; MBC), and blank controls (PBS instead of extracts) to the respective wells. Next, the bacterial suspension was added to each well. After 15 min of incubation in a prepared plate reader (dark conditions immediately before measurements), fluorescence was measured with the filter set to an excitation wavelength of 350 nm and an emission wavelength of 410 nm as a function of time, until there was no further increase in fluorescence. As outer membrane permeability increased by the addition extracts, NPN incorporated into the membrane caused an increase in fluorescence. The percentage of NPN uptake was calculated using the following equation [54]:

% NPN uptake = (F_obs_ − F_0_)/(F_100_ − F_0_) × 100,

where F_obs_ is the observed fluorescence at MBC extract concentration, F_0_ the fluorescence of the blank, and F_100_ is the fluorescence of gramicidin (positive control).

### 4.6. Checkerboard Arrays for Planktonic Bacteria (Fractional Inhibitory Concentration Index)

The demonstration of the interactions between extracts and antibiotics is important due to the possibility of synergy detection. The presence of such effects may reduce the therapeutic dose of the antibiotic while strengthening the therapeutic effect, reducing undesirable side effects and bacterial resistance to the antibiotics. To evaluate such interactions, the checkerboard assay was applied.

The checkerboard arrays method was described by Turecka et al. [55]. MH broth was used in the studies. We tested the antibiotics cefoxitin and amikacin in the range of 250 to 15.6 µg/mL and extracts in the range of 390 to 3125 µg/mL. Bacteria were cultured for 48 h in aerobic conditions. After incubation, the turbidity of the samples was visually determined, and the MICs of the antibiotic and the test compound were determined and subsequently substituted into the formula to calculate the Fractional Inhibitory Concentration Index (FICI).

$$\mathrm{FIC}\;\mathrm{of}\;\mathrm{extract}\;\left(\mathrm{FICE}\right)=\frac{MIC\;\mathrm{of}\;\mathrm{extract}\;\mathrm{in}\;\mathrm{combination}}{\mathrm{MIC}\;\mathrm{of}\;\mathrm{extract}\;\mathrm{alone}}$$

$$\mathrm{FIC}\;\mathrm{of}\;\mathrm{antibiotic}\;\left(\mathrm{FICA}\right)=\frac{MIC\;\mathrm{of}\;\mathrm{antibiotic}\;\mathrm{in}\;\mathrm{combination}}{\mathrm{MIC}\;\mathrm{of}\;\mathrm{antibiotic}\;\mathrm{alone}}$$

FICI=FICE+FICA

Then, FICI was interpreted as follows: synergism (FICI < 0.5), semi-synergism (0.5 < FICI < 1.0), additive effect (1 < FICI < 4), and antagonism (FICI > 4) [55].

### 4.7. Statistical Analysis

All experiments were performed at least three times. The intergroup differences were estimated by one- or two-way analysis of variance using Microsoft Excel 2010. All data were additionally analyzed by STATISTICA ANOVA v. 13.3. The distribution of normality of continuous variables was calculated using the Shapiro–Wilk test. The data are presented as mean and standard deviation (±SD). A *p* value was considered as statistically significant when it was less than 0.05.

## 5. Conclusions

In this study, the activity of ethanolic and aqueous extracts of Rubus caesius leaves (L_H2O_, L_EtOH_) and stems (S_H2O_, S_EtOH_) is continued. The activity of the extracts against reference and clinical strains of S. aureus and CNS was tested. The results obtained showed that L_EtOH_ was the most active extract, L_H2O_ and S_EtOH_ extracts were less active, and S_H2O_ extract showed the weakest activity against bacteria. The studies showed that Rubus caesius extracts showed antimicrobial activity against bacteria in biofilms. L_EtOH_ extract most strongly inhibited biofilm formation by clinical strains MRSA and MRCNS at all tested concentrations. In turn, S_EtOH_ also inhibited biofilm formation, but had a stronger effect on MRSA strains than on MRCNS. Furthermore, L_H2O_ extract showed the weakest antibacterial activity against the stage of biofilm formation in both groups of bacteria. All tested extracts showed a weak inhibitory effect on the development of already formed biofilms. The effect of Rubus caesius extracts on the cytoplasmic membrane was demonstrated. DiSC_3_(5) and NPN probes were used in these experiments. The studies showed that the composition of components contained in the S_EtOH_ extract caused the strongest effect of depolarization of the cell membrane of *S. aureus* and CNS strains, including MR strains. On the other hand, the L_EtOH_ extract was less active and only affected *S. aureus* strains, including the MRSA strain. Moreover, the interaction between Rubus caesius extracts and antibiotics was demonstrated for the first time. In the case of the L_EtOH_ extract, a synergistic effect was observed in combination with amikacin and cefoxitin. The same effect was observed for the S_EtOH_ extract and cefoxitin, but an additive effect was observed for amikacin. The aqueous extracts showed synergism (L_H2O_) and additivity with cefoxitin and antagonism with amikacin. It is worth noting that the extracts caused a decrease in the MIC values of the antibiotics used from 8- to 16-fold, and the MIC values of the extracts decreased from 4- to 8-fold. It seems that the presence of larger amounts of hyperoside, tiliroside, isoquercetin, rutin, quercetin, isokaemferide and catechins in the ethanol extracts than in the aqueous extracts is of crucial importance in the activity against *S. aureus*, including MRSA or MRCNS. The extracts can be used as preparations preventing infections and biofilm development by S. aureus strains, as well as to reduce the effective dose of selected antibiotics administered during the treatment of infections, which has therapeutic significance.

## Figures and Tables

**Table 1:** Minimum inhibitory concentrations (MICs) [mg/mL] and minimum bactericidal concentrations (MBCs) [mg/mL] of extracts and gramicidin for reference bacterial strains.

Bacteria	Gramicidin		Extracts [mg/mL]							
			S_EtOH_		S_H2O_		L_EtOH_		L_H2O_	
	MIC	MBC	MIC	MBC	MIC	MBC	MIC	MBC	MIC	MBC
***S. AUREUS*** **ATCC6538**	0.03125 ±0.15	>0.125	0.16 ±0.40	10 ±0.58	0.16 ±0.40	5 ±0.32	0.16 ±0.40	10 ±0.55	0.16 ±0.20	5 ±0.54
***S. EPIDERMIDIS*** **ATCC14990**	0.00196 ±0.20	0.125 ±0.50	0.625 ±0.40	5 ±0.52	0.04 ±0.15	>10	0.625 ±0.50	5 ±0.49	10 ±0.54	10 ±0.65
***S. AUREUS*** **ATCC43300** **MRSA**	0.125 ±0.14	>0.125	0.625 ±0.40	5 ±0.38	0.3125 ±0.20	10 ±0.10	0.625 ±0.20	5 ±0.38	0.16 ±0.42	5 ±0.12
**MRSA 12673**	0.03125 ±0.22	>0.125	0.39 ±0.10	1.56 ±0.40	1.56 ±0.50	>12.5	0.78 ±0.50	12.5 ±0.54	0.39 ±0.10	3.125 ±0.40
**MRSA 15732**	0.06250 ±0.24	0.125 ±0.50	1.56 ±0.60	12.5 ±0.65	0.78 ±0.10	>12.5	0.78 ±0.40	6.25 ±0.40	0.78 ±0.30	12.5 ±0.40
**MRSA 12677**	>0.250	>0.125	0.39 ±0.50	6.25 ±0.52	3.125 ±0.51	>12.5	1.56 ±0.64	6.25 ±0.43	0.78 ±0.25	6.25 ±0.55
**MRSA 12753**	0.0625 ±0.17	>0.125	0.78 ±0.52	0.78 ±0.48	0.78 ±0.35	>12.5	1.56 ±0.52	1.56 ±0.12	1.56 ±0.56	3.125 ±0.18
**MRSA 13251**	0.01563 ±0.20	0.125	0.39 ±0.25	1.56 ±0.44	3.125 ±0.52	>12.5	0.78 ±0.45	3.125 ±0.52	0.78 ±0.56	1.56 ±0.23
**MRSA 18532**	0.03125 ±0.18	>0.125	3.125 ±0.45	3.125 ±0.58	12.5 ±0.56	12.5 ±0.10	0.78 ±0.61	1.56 ±0.48	0.78 ±0.43	1.56 ±0.65
**MRSA 21804**	0.03125 ±0.21	>0.125	0.39 ±0.18	0.78 ±0.41	>12.5	>12.5	0.78 ±0.65	1.56 ±0.50	0.78 ±0.68	1.56 ±0.58
**MRSE 13199**	0.00024 ±0.12	0.01563 ±0.18	0.78 ±0.28	0.78 ±0.65	0.78 ±0.24	>12.5	1.56 ±0.54	3.125 ±0.22	0.78 ±0.12	0.78 ±0.48
**MRCNS 16000**	>0.250	>0.125	0.39 ±0.65	0.78 ±0.54	1.56 ±0.35	>12.5	0.78 ±0.58	1.56 ±0.32	0.78 ±0.25	1.56 ±0.56
**MRCNS 16248**	0.00098 ±0.21	0.0625 ±0.22	0.78 ±0.56	1.56 ±0.54	>12.5	>12.5	1.56 ±0.23	1.56 ±0.45	3.125 ±0.21	6.25 ±0.25

S_EtOH_—stem ethanol extract, S_H2O_—stem water extract, L_EtOH_—leaf ethanol extract, L_H2O_—leaf water extract. The results are presented as mean values ± standard deviation (±SD) from three independent experiments.

**Table 2:** Effect of *R. caesius* extracts on membrane potentials of selected strains of bacteria.

	*S. aureus* ATCC6538	*S. epidermidis* ATCC14990	MRSA15732	MRCNS13199
S_EtOH_	298.58 ± 0.15	113.75 ± 0.21	62.24 ± 0.21	141.43 ± 0.10
S_H2O_	−1.85 ± 0.10	−6.90 ± 0.11	−0.78 ± 0.23	−33.93 ± 0.12
L_EtOH_	165.03 ± 0.12	43.97 ± 0.34	73.00 ± 0.12	−11.28 ± 0.23
L_H2O_	−0.38 ± 0.23	−12.66 ± 0.42	−14.65 ± 0.10	−59.63 ± 0.25

The results are presented as mean values ± standard deviation (±SD) from three independent experiments.

**Table 3:** FICI extract–antibiotic values for the tested strains.

Strains	Antibiotic	MIC Antibiotic [mg/mL]		*R. caesius* Extract	MIC Extract [mg/mL]		FICI	Outcome
		Alone	Comb.		Alone	Comb.		
MRSA12673	amikacin	0.003906	0.001953	S_EtOH_	1.563	0.781	1.000	additivity
			0.015625	S_H2O_	3.125	3.125	4.995	antagonism
			0.000490	L_EtOH_	6.250	0.781	0.250	synergy
			0.015625	L_H2O_	3.125	1.563	4.495	antagonism
	cefoxitin	0.250000	0.015625	S_EtOH_	1.563	0.390	0.281	synergy
			0.500000	S_H2O_	6.250	6.250	2.000	additivity
			0.015625	L_EtOH_	3.125	0.781	0.313	synergy
			0.015625	L_H2O_	0.390	0.195	0.563	semi-synergy
MRSA15732	amikacin	0.003906	0.001953	S_EtOH_	1.563	0.781	1.000	additivity
			0.062500	S_H2O_	12.500	12.500	17.000	antagonism
			0.001953	L_EtOH_	6.250	1.563	0.750	semi-synergy
			0.031250	L_H2O_	1.563	1.563	9.000	antagonism
	cefoxitin	0.250000	0.062500	S_EtOH_	1.563	0.781	0.750	semi-synergy
			0.125000	S_H2O_	6.250	3.125	1.000	additivity
			0.031250	L_EtOH_	6.250	0.781	0.250	synergy
			0.031250	L_H2O_	3.125	0.391	0.250	synergy

Comb.—combination: *R. caesius* extract + antibiotic or antibiotic + *R. caesius* extract.

## Data Availability

Data are contained within the article.

## Abbreviations

The following abbreviations are used in this manuscript:

MRSA	methicillin-resistant *Staphylococcus aureus*
MRCNS	methicyllin-resistant coagulase-negative *Staphylococcus*
L_H2O_	aqueous extract of dewberry leaves
L_EtOH_	ethanolic extract of dewberry leaves
S_H2O_	aqueous extract of dewberry stems
S_EtOH_	ethanolic extract of dewberry stems
ESKAPE	is an acronym comprising the scientific names of six highly virulent and antibiotic-resistant bacterial pathogens including: *Enterococcus faecium*, *Staphylococcus aureus*, *Klebsiella pneumoniae*, *Acinetobacter baumannii*,
MIC	minimum inhibitory concentrations
MBC	minimum bactericidal concentrations
FICI	Fractional Inhibitor Concentration Index
ATCC	American Type Culture Collection
PCM	*Polish Collection of Microorganisms*
CFU	colony-forming unit

## References

[1] Baran A., Kwiatkowska A., Potocki L. (2023). Antibiotics and Bacterial Resistance—A Short Story of an Endless Arms Race. Int. J. Mol. Sci..

[2] Gajdács M., Albericio F. (2019). Antibiotic Resistance: From the Bench to Patients. Antibiotics.

[3] Machado T.S., Pinheiro F.R., Andre L.S.P., Pereira R.F.A., Correa R.F., de Mello G.C., Ribeiro T.A.N., Penna B., Sachs D., Aguiar-Alves F. (2021). Virulence Factors Found in Nasal Colonization and Infection of Methicillin-Resistant *Staphylococcus aureus* (MRSA) Isolates and Their Ability to Form a Biofilm. Toxins.

[4] Derakhshan S., Navidinia M., Haghi F. (2021). Antibiotic susceptibility of humanassociated *Staphylococcus aureus* and its relation to agr typing, virulence genes, and biofilm formation. Infect. Dis..

[5] Mone N.S., Kamble E.E., Pardesi K.R., Satpute S.K. (2022). Antibacterial and Antibiofilm Potency of Menadione Against Multidrug-Resistant *S. aureus*. Curr. Microbiol..

[6] Kot B., Wierzchowska K., Piechota M., Grużewska A. (2020). Antimicrobial resistance patterns in methicillin-resistant *Staphylococcus aureus* from patients hospitalized during 2015–2017 in hospitals in Poland. Med. Princ. Pract..

[7] Hall C.W., Mah T.F. (2017). Molecular mechanisms of biofilm-based antibiotic resistance and tolerance in pathogenic bacteria. FEMS Microbiol. Rev..

[8] Tao J., Yan S., Zhou C., Liu Q., Zhu H., Wen Z. (2021). Total flavonoids from *Potentilla kleiniana* Wight et Arn inhibits biofilm formation and virulence factors production in methicillin-resistant *Staphylococcus aureus* (MRSA). J. Ethnopharmacol..

[9] Paul P., Chakraborty P., Chatterjee A., Sarker R.K., Dastidar D.G., Kundu T., Sarkar N., Das A., Tribedi P. (2021). 1,4-Naphthoquinone accumulates reactive oxygen species in *S. aureus*: A promising approach towards effective management of biofilm threat. Arch. Microbiol..

[10] Salaheen S., Peng M., Joo J., Teramoto H., Biswas D. (2017). Eradication and Sensitization of Methicillin Resistant *Staphylococcus aureus* to Methicillin with Bioactive Extracts of Berry Pomace. Front. Microbiol..

[11] Welia A.M., Al-Saadi H.S., Al-Fudhaili R.S., Hossain A., Putit Z.B., Jasim M.K. (2020). Cytotoxic and antimicrobial potential of different leaves extracts of R. fruticosus used traditionally to treat diabetes. Toxicol. Rep..

[12] Aguilera-Correa J.J., Nohynek L., Alakomi H.-L., Esteban J., Oksman-Caldentey K.-M., Puupponen-Pimiä R., Kinnari T.J., Perez-Tanoira R. (2023). Reduction of methicillin-resistant *Staphylococcus aureus* biofilm growth and development using arctic berry extracts. Front. Cell. Infect. Microbiol..

[13] Saeed A. (2022). Antibacterial effect of *Rubus chingii* flower extract against multidrug-resistant bacteria. Bioinformation.

[14] Zhang Y., Chen J., Wang L., Cao J., Xu L. (2016). Chemical composition and biological activities of the essential oil from *Rubus pungens* var. *oldhamii*. Nat. Prod. Res..

[15] Krauze-Baranowska M., Głód D., Kula M., Majdan M., Hałasa R., Matkowski M., Kozłowska W., Kawiak A. (2014). Chemical composition and biological activity of *Rubus idaeus* shoots—A traditional herbal remedy of Eastern Europe. BMC Complement. Altern. Med..

[16] Krauze-Baranowska M., Majdan M., Hałasa R., Głod D., Kula M., Feckac I., Orzeł A. (2014). The antimicrobial activity of fruits from some cultivar varieties of *Rubus idaeus* and *Rubus occidentalis*. Food Funct..

[17] Hering A., Stefanowicz-Hajduk J., Hałasa R., Olech M., Nowak R., Kosinski P., Ochocka J.R. (2022). Polyphenolic Characterization, Antioxidant, Antihyaluronidase and Antimicrobial Activity of Young Leaves and Stem Extracts from *Rubus caesius* L. Molecules.

[18] Oulahal N., Degraeve P. (2022). Phenolic-Rich Plant Extracts With Antimicrobial Activity: An Alternative to Food Preservatives and Biocides?. Front. Microbiol..

[19] Benfield A.H., Henriques S.T. (2020). Mode-of-Action of Antimicrobial Peptides: Membrane Disruption vs. Intracellular Mechanisms. Front. Med. Technol..

[20] Grabek-Lejko D., Miłek M., Sidor E., Puchalski C., Dzugan M. (2022). Antiviral and Antibacterial Effect of Honey Enriched with *Rubus* spp. as a Functional Food with Enhanced Antioxidant Properties. Molecules.

[21] De Santis D., Carbone K., Garzoli S., Laghezza Masci V., Turchetti G. (2022). Bioactivity and Chemical Profile of *Rubus idaeus* L. Leaves Steam-Distillation Extract. Foods.

[22] Krzepiłko A., Prażak R., Święciło A. (2021). Chemical Composition, Antioxidant and Antimicrobial Activity of Raspberry, Blackberry and Raspberry-Blackberry Hybrid Leaf Buds. Molecules.

[23] Grabek-Lejko D., Wójtowicz K. (2014). Comparison of antibacterial and antioxidant properties of fruits and leaves of blackberry (*Rubus plicatus*) and raspberry (*Rubus idaeus*). J. Microbiol. Biotechnol. Food Sci..

[24] Quave C.L., Estevez-Carmona M., Compadre C.M., Hobby G., Hendrickson H.P., Beenken K.E., Smeltzer M.S. (2012). Ellagic acid derivatives from *Rubus ulmifolius* inhibit *Staphylococcus aureus* biofilm formation and improve response to antibiotics. PLoS ONE.

[25] Quavea C.L., Plano L.R.W., Pantuso T., Bennett B.C. (2008). Effects of extracts from Italian medicinal plants on planktonic growth, biofilm formation and adherence of methicillin-resistant *Staphylococcus aureus*. J. Ethnopharmacol..

[26] Reygaert W.C. (2014). The antimicrobial possibilities of green tea. Front. Microbiol..

[27] Górniak I., Bartoszewski R., Króliczewski J. (2019). Comprehensive review of antimicrobial activities of plant flavonoids. Phytochem. Rev..

[28] Sanver D., Murray B.S., Sadeghpour A., Rappolt M., Nelson A.L. (2016). Experimental modeling of flavonoid-biomembrane interactions. Langmuir.

[29] Zhang L., Kong Y., Wu D., Zhang H., Wu J., Chen J., Ding J., Hu L., Jiang H., Shen X. (2008). Three flavonoids targeting the beta-hydroxyacyl-acyl carrier protein dehydratase from *Helicobacter pylori*: Crystal structure characterization with enzymatic inhibition assay. Protein Sci..

[30] Wu D., Kong Y., Han C., Chen J., Hu L., Jiang H., Shen X. (2008). DAlanine: D-alanine ligase as a new target for the flavonoids quercetin and apigenin. Int. J. Antimicrob. Agents.

[31] Suriyanarayanan B., Shanmugam K., Santhosh R. (2013). Synthetic quercetin inhibits mycobacterial growth possibly by interacting with DNA gyrase. Rom. Biotechnol. Lett..

[32] Chinnam N., Dadi P.K., Sabri S.A., Ahmad M., Kabir M.A., Ahmad Z. (2010). Dietary bioflavonoids inhibit Escherichia coli ATP synthase in a differential manner. Int. J. Biol. Macromol..

[33] Raheem N., Straus S.K. (2019). Mechanisms of Action for Antimicrobial Peptides With Antibacterial and Antibiofilm Functions. Front. Microbiol..

[34] Vikram A., Jayaprakasha G.K., Jesudhasan P.R., Pillai S.D., Patil B.S. (2010). Suppression of bacterial cell–cell signalling, biofilm formation and type III secretion system by citrus flavonoids. J. Appl. Microbiol..

[35] Ouyang J., Sun F., Feng W., Sun Y., Qiu X., Xiong L., Liu Y., Chen Y. (2016). Quercetin is an effective inhibitor of quorum sensing, biofilm formation and virulence factors in Pseudomonas aeruginosa. J. Appl. Microbiol..

[36] Symeonidis A., Marangos M., Priti D.R. (2012). Iron and microbial growth. Insight and Control of Infectious Disease in Global Scenario.

[37] Fontaine B.M., Nelson K., Lyles J.T., Jariwala P.B., García-Rodriguez J.M., Quave C.L., Weinert E.E. (2017). Identification of Ellagic Acid Rhamnoside as a Bioactive Component of a Complex Botanical Extract with Anti-biofilm Activity. Front. Microbiol..

[38] Cushnie T.P., Taylor P.W., Nagaoka Y., Uesato S., Hara Y., Lamb A.J. (2008). Investigation of the antibacterial activity of 3-Ooctanoyl-(-)- epicatechin. J. Appl. Microbiol..

[39] Hałasa R., Mizerska U., Kula M., Krauze-Baranowska M. (2024). Screening Tests for the Interaction of *Rubus idaeus* and Rubus occidentalis Extracts with Antibiotics against Gram-Positive and Gram-Negative Human Pathogens. Antibiotics.

[40] Savedra M.J., Borges A., Dias C., Aires A., Bennett R.N., Rosa E.S., Simões M. (2010). Antimicrobial activity of phenolics and glucosinolate hydrolysis products and their synergy with streptomycin against pathogenic bacteria. Med. Chem..

[41] Wang S., Yao J., Zhou B., Yang J., Chaudry M.T., Wang M., Fenglin X., Li Y., Yin W. (2018). Bacteriostatic effect of quercetin as an antibiotic alternative in vivo and its antibacterial mechanism in vitro. J. Food Prot..

[42] Miklasińska-Majdanik M., Kępa M., Wojtyczka R.D., Idzik D., Wąsik T.J. (2018). Phenolic Compounds Diminish Antibiotic Resistance of *Staphylococcus aureus* Clinical Strains. Int. J. Environ. Res. Public Health.

[43] Marni S., D’Addariob C., Colacevichc A., Focardi S., Borghinic F., Santuccid A., Figurae N., Rossi C. (2009). Antimicrobial activity against *Helicobacter pylori* strains and antioxidant properties of blackberry leaves (*Rubus ulmifolius*) and isolated compounds. Int. J. Antimicrob. Agents.

[44] Kepa M., Miklasinska-Majdanik M., Wojtyczka R.D., Idzik D., Korzeniowski K., Smolen-Dzirba J., Wasik T.J. (2018). Antimicrobial Potential of Caffeic Acid against *Staphylococcus aureus* ClinicalStrains. Biomed. Res. Int..

[45] Siriwong S., Thumanu K., Hengpratom T., Eumkeb G. (2015). Synergy and mode of action of ceftazidime plus quercetin or luteolin on *Streptococcus pyogenes*. Evid. Based Complement. Alternat. Med..

[46] Zhao W.H., Hu Z.Q., Okubo S., Hara Y., Shimamura T. (2001). Mechanism of synergy between epigallocatechin gallate and beta-lactams against methicillin-resistant *Staphylococcus aureus*. Antimicrob. Agents Chemother..

[47] Zhao W.H., Hu Z.Q., Okubo S., Hara Y., Shimamura T. (2002). Inhibition of penicillinase by epigallocatechin gallate resulting in restoration of antibacterial activity of penicillin against penicillinase-producing *Staphylococcus aureus*. Antimicrob. Agents Chemother..

[48] Novy P., Rondevaldova J., Kourimska L., Kokoska L. (2013). Synergistic interactions of epigallocatechin gallate and oxytetracycline against various drug resistant *Staphylococcus aureus* strains in vitro. Phytomedicine.

[49] Hu Z.Q., Zhao W.H., Asano N., Yoda Y., Hara Y., Shimamura T. (2002). Epigallocatechin gallate synergistically enhances the activity of carbapenems against methicillin-resistant *Staphylococcus aureus*. Antimicrob. Agents Chemother..

[50] Bułakowska A., Sławiński J., Hałasa R., Hering A., Gucwa M., Ochocka J.R., Stefanowicz-Hajduk J. (2023). An In Vitro Antimicrobial, Anticancer and Antioxidant Activity of N–[(2–Arylmethylthio)phenylsulfonyl] cinnamamide Derivatives. Molecules.

[51] Cheng M., Huang J.X., Ramu S., Butler M.S., Cooper M.A. (2014). Ramoplanin at Bactericidal Concentrations Induces Bacterial Membrane Depolarization in *Staphylococcus aureus*. Antimicrob. Agents Chemother..

[52] Cheng J.T., Hale J.D., Elliot M., Hancock R.E., Straus S.K. (2009). Effect of membrane composition on antimicrobial peptides aurein 2.2 and 2.3 from Australian southern bell frogs. Biophys. J..

[53] Ombredane A.S., Martins N.O., de Souza G.M.V., Araujo V.H.S., Szlachetka Í.O., da Silva S.W., da Rocha M.C.O., Oliveira A.S.d., Holanda C.A., Romeiro L.A.S. (2024). Combinatory Effect of Pequi Oil (Caryocar brasiliense)-Based Nanoemulsions Associated to Docetaxel and Anacardic Acid (*Anacardium occidentale*) in Triple Negative Breast Cancer Cells In Vitro. Pharmaceutics.

[54] Jacob B., Kim Y., Hyun J.K., Park I.S., Bang J.K., Shin S.Y. (2014). Bacterial killing mechanism of sheep myeloid antimicrobial peptide-18 (SMAP-18) and its Trp-substituted analog with improved cell selectivity and reduced mammalian cell toxicity. Amino Acids.

[55] Turecka K., Chylewska A., Kawiak A., Waleron K.F. (2018). Antifungal Activity and Mechanism of Action of the Co(III) Coordination Complexes With Diamine Chelate Ligands Against Reference and Clinical Strains of *Candida* spp. Front. Microbiol..

